# Momentary and Longitudinal Effects of Loneliness on Dementia Caregiver Well-Being

**DOI:** 10.1093/geroni/igaf122.4254

**Published:** 2025-12-31

**Authors:** Christopher Fagundes, Pauline Goodson, Vincent D Lai, Daniel Argueta, Paula Lanternier, Valentina Maza Santibañez, Kelly N Brice, Brenda Zarazua-Osorio

**Affiliations:** Rice University, Houston, Texas, United States; Rice University, Houston, Texas, United States; Rice University, Houston, Texas, United States; Rice Univdrsity, Houston, Texas, United States; Rice University, Houston, Texas, United States; Rice University, Houston, Texas, United States; Rice University, Houston, Texas, United States; Rice University, Houston, Texas, United States

## Abstract

Spousal caregivers of individuals with Alzheimer’s disease and Alzheimer’s disease-related dementias (AD/ADRD) often experience profound loneliness, which contributes to persistent psychological distress and elevated risk for morbidity and mortality. Yet the mechanisms through which loneliness shapes their emotional well-being and caregiving outcomes remain unclear. Guided by stress process and attachment frameworks, we tested both momentary and longitudinal effects of loneliness in AD/ADRD spousal caregivers. Participants (N = 69; mean age = 71.5 years; 64% female; 62% White) completed ecological momentary assessments of loneliness and affect, along with baseline and follow-up surveys of caregiver burden, depressive symptoms, and anticipatory grief. Multilevel models showed that within-person increases in loneliness predicted greater depressed affect (b = 0.72, p < .001), controlling for demographics. Longitudinally, higher baseline loneliness predicted increases in caregiver burden (b = 0.21, p = .02) and anticipatory grief (b = 0.01, p = .01), but not depressive symptoms, beyond prior levels. Moderation analyses indicated that attachment anxiety heightened vulnerability to depressed affect, whereas attachment avoidance buffered the within-person daily link between loneliness and depressed affect. Findings demonstrate that loneliness functions as both a dynamic daily process and a long-term vulnerability factor in AD/ADRD spousal caregiving. By identifying attachment insecurity as a risk amplifier, this work provides mechanistic insight into when and for whom loneliness is most detrimental and highlights loneliness as a promising intervention target to reduce burden, mitigate anticipatory grief, and promote resilience among AD/ADRD spousal caregivers.

